# Effects of Remote Ischemic Preconditioning in Patients Undergoing Off-Pump Coronary Artery Bypass Graft Surgery

**DOI:** 10.3389/fphys.2019.00495

**Published:** 2019-04-29

**Authors:** Huilin Wang, Yi Lyu, Qingwu Liao, Lin Jin, Liying Xu, Yan Hu, Ying Yu, Kefang Guo

**Affiliations:** ^1^Department of Anesthesiology, Zhongshan Hospital, Fudan University, Shanghai, China; ^2^Department of Anesthesiology, Yunnan Baoshan Anli Hospital, Baoshan, China

**Keywords:** off-pump coronary artery bypass graft surgery, remote ischemic preconditioning, myocardial protection, outcome, protective therapeutic approach

## Abstract

**Purpose:**

This study aimed to evaluate effects of remote ischemic preconditioning (RIPC) on myocardial injury in patients undergoing off-pump coronary artery bypass graft surgery (OPCABG).

**Methods:**

Sixty-five patients scheduled for the OPCABG were randomly assigned to control (*n* = 32) or RIPC group (*n* = 33). All patients received general anesthesia. Before the surgical incision, RIPC was induced on an upper limb with repeated 5-min ischemia and 5-min reperfusion for four times. Blood samples were collected from right internal jugular vein. Plasma levels of IL-6, IL-8, IL-10, TNF-α, cTnT, HFABP, IMA, and MDA were detected at pre-operatively and 0, 6, 18, 24, 48, 72, and 120 h after the surgery. Left internal mammary artery (LIMA) and great saphenous vein (GSV) was cut into 2–3 mm for Western blot analysis of Hif-1α.

**Results:**

In the present study, RIPC treatment significantly reduced plasma levels of cardiac troponin T (*p* < 0.05), heart-type fatty acid binding protein (*p* < 0.05), ischemia modified albumin (*p* < 0.05), malondialdehyde (*p* < 0.05), as well as plasma levels of pro-inflammatory cytokines including IL-6, IL-8, and TNF-α (*P* < 0.05, respectively). RIPC treatment significantly increased hypoxia-inducible factor-1α (*p* < 0.05) expression as well. Mechanical ventilation time for postoperative patients was shortened in RIPC group than those in control group (17.4 ± 3.8 h vs. 19.7 ± 2.9 h, respectively, *p* < 0.05).

**Conclusion:**

RIPC by upper limb ischemia shortens mechanical ventilation time in patients undergoing OPCABG. RIPC treatment reduces postoperative myocardial enzyme expression and pro-inflammatory cytokine production. RIPC is a protective therapeutic approach in the coronary artery bypass graft surgery.

## Introduction

With the change of lifestyle and eating habits, the incidence of coronary artery disease (CAD) in China increased gradually (reported by Xue and Xu, 2017). Those who with multiple CAD need surgery. Off-pump coronary artery bypass graft (OPCABG) is the preferred surgical procedure. Even so, myocardial ischemia-reperfusion injury often occurs after OPCABG and this can not be avoided completely (Chowdhury et al., 2008). Thus, great efforts should be made to alleviate myocardial injury. Remote ischemic preconditioning (RIPC), a non-invasive and operable intervention in the clinic, has drawn the attention of clinicians in recent years.

The phenomenon of RIPC was first reported by Przyklenk et al. (1993) in the end of last century. After several years, the paradigm of “cardioprotection at a distance” by ischemic conditioning was quickly extended to other tissues and organs and to longer distances from the heart (Heusch, 2018). The underlying mechanisms probably include a release of transferable humoral from the perfused tissue and neuronal reflexes (Heusch et al., 2015).

It has been reported that RIPC could be a potential protective approach for perioperative complication (Cheung et al., 2006; Hausenloy et al., 2007). In patients undergone surgical coronary revascularization under isoflurane anesthesia, RIPC was confirmed to improve clinical outcome (Thielmann et al., 2013; Kleinbongard et al., 2018). However, the benefits from RIPC during cardiovascular surgery were not confirmed in other two large phase III trials (Hausenloy et al., 2015; Meybohm et al., 2015). Meanwhile, the effects of RIPC on myocardial injury as well as clinical outcome in patients undergoing OPCABG are inconclusive. In the present study, we conducted a randomized clinical trial on patients undergoing OPCABG. To augment the protective effect of RIPC, RIPC was given as upper limb ischemia. Myocardial injury was assessed by measuring plasma cTnT, IMA, HFABP level at baseline and after operation. Inflammatory reaction (IL-6, IL-8, TNF-α, and IL-10), Hif-1α and oxidation index (MDA) were also examined. Clinical parameters analysis was used to assess short-term prognosis.

## Materials and Methods

### Patients

The present study was approved by Ethnics Committee, Zhongshan Hospital, Fudan University^1^. All patients recruited in the present study were signed up with written consent before enrollment. Exclusion criteria of the present study include age >80 years, major combined surgery (such as valve surgery), myocardial infarction in the last 28 days, severe infection in the last 7 days, severe hepatic, renal, pulmonary or hematological disease; use of an inotropic agent or a mechanical assist device; left ventricular ejection fraction less than 40%; or peripheral vascular disease affecting upper limbs. Patients were randomly assigned to control or RIPC group using a computer-generated random list after they entering the operating room. Both surgeons and anesthesiologists were blinded to the assignments.

### Anesthesia Method

Patients were given 40% concentration of oxygen inhalation by mask, right internal jugular vein catheterization and left radial artery catheterization were completed by anesthetist under ECG and pulse oxygen saturation monitoring. Anesthesia was induced with i.v. midazolam (0.05 mg/kg), etomidate (0.3–0.4 mg/kg), sufentanil (0.4–0.5 μg/kg), and rocuronium (0.9–1.2 mg/kg). The trachea was intubated with a tracheal tube (7# for women and 7.5# for men) under the exposure of visual laryngoscope and lungs were mechanically ventilated with 50% concentration of oxygen to maintain an end-tidal carbon dioxide tension of 35–40 mmHg. Anesthesia was maintained by sevoflurane (0.8–1.3 MAC) to achieve a bispectral index of 40–60. Rocuronium (0.2–0.3 mg/kg) and sufentanil (0.3–0.4 μg/kg) were added according to the clinical situation.

### RIPC Procedure

Remote ischemic preconditioning was induced with repeated 5-min ischemia and 5-min reperfusion on the upper limb for four times. By using a blood pressure cuff inflation, patients in RIPC group were exposed to a pressure 40 mmHg higher than the systolic arterial pressure, whereas control group had sham placement of the pressure cuff without inflation. RIPC procedure was performed right after the end of anesthesia induction.

### Surgical Method

Incision began after the whole RIPC procedure had been done. Anastomoses were constructed using an intracoronary shunt (Medtronic, Minneapolis, MN, United States). The left internal mammary artery (LIMA) and the great saphenous vein (GSV) were harvested primarily. Depending on the patients’ target vessel characteristics, other grafts such as the right internal mammary artery and the left radial artery were used in addition. Papaverine solution was used to avoid grafts vasospasm. Heparin was used (1 mg/kg per patient primarily) to achieve ACT value over 280 s.

### Blood Sample Collection and Analysis

Blood samples were collected from right internal jugular vein pre-operatively (Preop), and after the surgery (Postop 0, 6, 18, 24, 48, 72, and 120 h). After centrifuged at 1000 × *g* for 10 min, plasma samples were frozen at −80°C for later analysis. Plasma levels of inflammatory cytokines IL-6, IL-8, IL-10, and TNF-α were measured with a Luminex protein suspension array system (Bio-plex 200; Bio-Rad) according to manufacturer’s instructions. Plasma levels of cTnT (Human cTnT ELISA Kit, MBS2508285, MyBioSource, San Diego, CA, United States), HFABP (Human FABP3 DuoSet ELISA, DY1678, R&D) IMA (Human IMA ELISA Kit, MBS2515981, MyBioSource, San Diego, CA, United States), and MDA (Human MDA assay kit, A003-2, Jiancheng Inc., Jiangsu, China) were measured according to manufacturer’s instruction.

### Tissue Protein Extraction and Determination by Western Blot Analysis

After dissociation, LIMA and GSV was cut into 2–3 mm for Western blot analysis of Hif-1α (*n* = 13 in each group). The tissue samples were homogenized in Radio Immunoprecipitation Assay (RIPA, Cell Signaling, Boston, MA, United States). After centrifuged with 12000 rpm at 4°C for 10 min, supernatant was collected. Protein concentration were measured with bicinchoninic acid method (Bio-Rad, Hercules, CA, United States). The membranes were incubated with primary antibodies Hif-1α (1:1000; H1alpha67; Abcam, Cambridge, MA, United States) and GAPDH (1:2000; A2228; Sigma-Aldrich, St. Louis, MO, United States) at 4°C for overnight and corresponding secondary antibody at room temperature for 2 h. The detected bands were visualized with an ECL detection kit (Pierce, IL, United States).

### Statistical Analysis

As showed in reference, prolonged ICU stay is a powerful predictor of adverse outcome after cardiac surgery (Mahesh et al., 2012). With a two-sided significance level α of 0.05 and study power at 80%, it was estimated that 32 patients would be required per group in order to reach the conditions that RIPC could reduce the ICU stay time by 3 h (Sakpal, 2010). All datas are presented as the means ± SD. The comparison of enumerated data between the treatment groups was conducted using the chi-square test, while an unpaired *t*-test was applied to compare measurement data where appropriate. Plasma concentrations of cTnT, HFABP, IMA, MDA, and inflammatory cytokines were analyzed by 2-way (group, time) ANOVA for repeated measures followed by Fisher’s *post hoc* tests. SPSS version 19.0 for Windows (IBM Corporation, Armonk, NY, United States) were used for the statistical analyses. A value of *P* < 0.05 was considered as statistically significantly different.

## Results

The characteristics of patients in the two groups were comparable regarding their gender, age and body weight (Table 1). There were no significant differences in operation time (including bridging vessels dissociation time, bridging vessels anastomosis time and duration of surgery) between two groups. Patients in the control group had a longer mechanical ventilation time than those in RIPC group (control vs. RIPC: 19.7 ± 2.9 h vs. 17.4 ± 3.8 h, *p* < 0.05), while had similar amount of time staying the Intensive Care Unit (*p* > 0.05) and in regular wards (*p* > 0.05) (Table 2). Incidences of post-operative complications including hospital death were comparable in the two groups as well. However, no significant differences were observed between the two groups during the period of observation (Table 3).

**TABLE 1:** Patient characteristics.

Variable	Control group (*n* = 32)	RIPC group (*n* = 33)	*P*
Age (years)	59.8 ± 9.2	61.3 ± 8.7	0.587
Male	23 (72)	25 (75)	0.251
Weight (kg)	64.5 ± 10.3	66.7 ± 9.8	0.733

*Data given as mean ± SD. RIPC, remote ischemic preconditioning. There were no significant differences in gender, age, and body weight between the two groups.*

**TABLE 2:** Operative characteristics and short-term outcome.

Variable	Control group (*n* = 32)	RIPC group (*n* = 33)	*P*
Bridging vessels dissociation time (min)	64.1 ± 18.8	68.4 ± 24.2	0.403
Bridging vessels anastomosis time (min)	100.3 ± 26.4	100.1 ± 44.6	0.987
Duration of surgery (min)	233.1 ± 36.8	235.1 ± 43.4	0.841
Mechanical ventilation time (h)	19.7 ± 2.9	17.4 ± 3.8	0.006*
ICU stay (d)	1.8 ± 1.1	1.7 ± 1.0	0.673
Hospital stay (d)	7.9 ± 2.3	7.2 ± 1.1	0.118

*Data given as mean ± SD. ICU, intensive care unit. Patients in the control group had a longer mechanical ventilation time than those in RIPC group. There were no significant differences in other operative characteristics, ICU stay or hospital stay between the two groups.*

**TABLE 3:** Incidences of post-operative complications.

Major postoperative complications	Control group (*n* = 32)	RIPC group (*n* = 33)	*P*
AKI, n (%)	4 (12.5)	4 (12.1)	0.948
Renal failure requires dialysis, n (%)	0 (0)	0 (0)	N/A
Abnormal heart rhythm, n (%)	5 (15.6)	2 (6.1)	0.339
Delirium, n (%)	1 (3.1)	0 (0)	0.492
Pneumonia, n (%)	5 (15.6)	3 (9.1)	0.672
Postoperative infection, n (%)	1 (3.1)	1 (3)	0.965
Mechanical ventilation >48 h, n (%)	0 (0)	0 (0)	N/A
Reoperation, n (%)	0 (0)	0 (0)	N/A
Stroke, n (%)	0 (0)	0 (0)	N/A
Hospital death, n (%)	0 (0)	0 (0)	N/A
30-day mortality, n (%)	0 (0)	0 (0)	N/A

*Data given as median (lower-upper quartiles), n (%). AKI, acute kidney injury. There were no significant differences between the two groups during the period of observation.*

### Myocardial Injury

Before the surgery, plasma levels of IMA in two groups were comparable. After the surgery, the IMA levels in control group were increased. RIPC treatment significantly reduced the IMA levels after 18 h (control vs. RIPC: 15.48 ± 6.60 ng/ml vs. 9.82 ± 3.61 ng/ml, *p* < 0.05) (Table 4).

**TABLE 4:** Myocardial injury factors (IMA and HFABP).

	IMA (ng/ml)		HFABP (ng/ml)	
	Con (*n* = 32)	RIPC (*n* = 33)	Con (*n* = 32)	RIPC (*n* = 33)
Preop	10.56 ± 3.03	11.17 ± 4.42	2.81 ± 2.26	2.66 ± 1.76
Postop 0 h	13.14 ± 4.75	11.63 ± 4.20	5.56 ± 2.99*	3.72 ± 2.03*Δ
Postop 6 h	15.65 ± 5.03*	10.55 ± 4.44	4.21 ± 2.40*^#^	2.58 ± 2.02^#^Δ
Postop 18 h	15.48 ± 6.60*	9.82 ± 3.61Δ	2.96 ± 2.22^#^▲	2.07 ± 1.62^#^

Data given as mean ± SD. HFABP, heart-type fatty acid binding protein. IMA, ischemia modified albumin. Preop, pre-operation. Postop, post-operation. Δ*P* < 0.05 vs. control group; *^#^*P* and ▲*P* < 0.05 vs. within group. Compared with Preop, *P < 0.05, Compared with Postop 0 h, ^#^*P* < 0.05, Compared with Postop 6 h, ▲*P* < 0.05.

Before the surgery, plasma levels of HFABP in two groups were comparable. After the surgery, HFABP levels in control group were transiently increased. RIPC treatment significantly reduced the HFABP levels at 0 h (control vs. RIPC: 5.56 ± 2.99 ng/ml vs. 3.72 ± 2.03 ng/ml, *p* < 0.05) and 6 h (control vs. RIPC: 4.21 ± 2.40 ng/ml vs. 2.58 ± 2.02 ng/ml, *p* < 0.05) (Table 4).

Before the surgery, plasma levels of cTnT in two groups were comparable. After the surgery, the cTnT level in both group were increased. RIPC treatment significantly reduced the cTnT levels after 120 h (control vs. RIPC: 0.273 ± 0.397 ng/ml vs. 0.108 ± 0.110 ng/ml, *p* < 0.05) (Table 5).

**TABLE 5:** Myocardial injury factors (cTnT).

	cTnT (ng/ml)	
	Con (*n* = 32)	RIPC (*n* = 33)
Preop	0.023 ± 0.029	0.018 ± 0.017
Postop 6 h	0.314 ± 0327*	0.200 ± 0.113*
Postop 24 h	0.462 ± 0.765*	0.213 ± 0.108*
Postop 48 h	0.532 ± 0.989	0.187 ± 0.131*
Postop 72 h	0.384 ± 0.695	0.144 ± 0.136*
Postop 120 h	0.273 ± 0.397*	0.108 ± 0.110*^#^▲Δ

Data given as mean ± SD. cTnT, cardiac troponin T. Δ*P* < 0.05 vs. control group; *^#^*P* and ▲*P* < 0.05 vs. within group. Compared with Preop, *P < 0.05, Compared with Postop 0 h, ^#^*P* < 0.05, Compared with Postop 6 h, ▲*P* < 0.05.

### Systemic Inflammatory Response

Before the surgery, both groups had comparable plasma levels of IL-6, IL-8, and TNF-α. After the surgery, plasma levels of IL-6, IL-8, and TNF-α were significantly increased in both groups. RIPC treatment significantly reduced the plasma levels of IL-6, IL-8 after 0 h (control vs. RIPC: 0.88 ± 0.39 ng/ml vs. 0.49 ± 0.34 ng/ml, *p* < 0.05) (control vs. RIPC: 1.41 ± 0.91 ng/ml vs. 0.78 ± 0.37 ng/ml, *p* < 0.05), 6 h (control vs. RIPC: 10.81 ± 6.61 ng/ml vs. 4.74 ± 2.62 ng/ml, *p* < 0.05) (control vs. RIPC: 3.99 ± 2.36 ng/ml vs. 2.24 ± 1.46 ng/ml, *p* < 0.05), and 18 h (control vs. RIPC: 7.78 ± 4.80 ng/ml vs. 2.89 ± 1.40 ng/ml, *p* < 0.05) (control vs. RIPC: 3.61 ± 2.00 ng/ml vs. 1.93 ± 0.91 ng/ml, *p* < 0.05), and TNF-α after 6 h (control vs. RIPC: 2.32 ± 0.79 ng/ml vs. 1.55 ± 0.87 ng/ml, *p* < 0.05) and 18 h (control vs. RIPC: 2.60 ± 1.04 ng/ml vs. 1.72 ± 0.91 ng/ml, *p* < 0.05).

Before the surgery, both groups had comparable levels of IL-10 in plasma. Plasma level of IL-10 were transiently, but significantly increased after the surgery (0 h) and then decreased. The plasma levels of IL-10 decreased largely in control group after 6 h (control vs. RIPC: 0.59 ± 0.34 ng/ml vs. 1.36 ± 0.80 ng/ml, *p* < 0.05) and 18 h compared with RIPC group (control vs. RIPC: 0.53 ± 0.32 ng/ml vs. 0.77 ± 0.45 ng/ml, *p* < 0.05) (Table 6).

**TABLE 6:** Systemic inflammatory response.

	IL-6 (ng/ml)		IL-8 (ng/ml)		TNF-1a (ng/ml)		IL-10 (ng/ml)	
	Con (*n* = 32)	RIPC (*n* = 33)	Con (*n* = 32)	RIPC (*n* = 33)	Con (*n* = 32)	RIPC (*n* = 33)	Con (*n* = 32)	RIPC (*n* = 33)
Preop	0.27±0.06	0.24±0.09	0.66±0.29	0.62±0.30	1.38±0.54	1.27±0.77	0.28±0.28	0.29±0.18
Postop 0 h	$0.88\pm 0.39^{*}$	$0.49\pm 0.34^{*}\,\Delta$	$1.41\pm 0.91^{*}$	0.78±0.37⁢Δ	1.56±0.68	1.33±0.47	$3.16\pm 2.38^{*}$	$3.11\pm 1.92^{*}$
Postop 6 h	$\,10.81\pm 6.61^{*}$	$4.74\pm 2.62^{*\#}\,\Delta$	$3.99\pm 2.36^{*\#}$	$2.24\pm 1.46^{*\#}\,\Delta$	$2.32\pm 0.79^{*\#}$	1.55±0.87⁢Δ	$0.59\pm 0.34^{*\#}$	$1.36\pm 0.80^{*\#}\,\Delta$
Postop 18 h	$\,7.78\pm 4.80^{*}$	$2.89\pm 1.40^{*\#}\,▲\,\Delta$	$3.61\pm 2.00^{*\#}\,▲$	$1.93\pm 0.91^{*\#}\,\Delta$	$2.60\pm 1.04^{*\#}$	$1.72\pm 0.91^{*\#}\,\Delta$	$0.53\pm 0.32^{*\#}$	$0.77\pm 0.45^{*\#}\,▲\,\Delta$

Data given as mean ± SD. IL, interleukin. TNF, tumor necrosis factor. Δ*P* < 0.05 vs. control group;*^#^*P* and ▲*P* < 0.05 vs. within group. Compared with Preop, *P < 0.05, Compared with Postop 0 h, ^#^*P* < 0.05, Compared with Postop 6 h, ▲*P* < 0.05.

### Oxidative Index

Before the surgery, both groups had comparable levels of MDA in plasma. Plasma level of MDA were significantly increased after the surgery. RIPC treatment significantly reduced the plasma levels of MDA at 0 h (control vs. RIPC: 66.07 ± 3.46 nmol/ml vs. 50.26 ± 3.22 nmol/ml, *p* < 0.05) (Table 7).

**TABLE 7:** Oxidative index.

	MDA (nmol/ml)	
	Con (*n* = 32)	RIPC (*n* = 33)
Preop	48.14 ± 4.14	47.25 ± 3.32
Postop 0 h	66.07 ± 3.46	50.26 ± 3.22*
Postop 6 h	79.30 ± 5.13	71.66 ± 4.87
Postop 18 h	54.51 ± 4.10	45.97 ± 3.10

Data given as mean ± SD. MDA, malonaldehyde. *P < 0.05 vs. control group.

### Hif-1α Protein Expression in LIMA and GSV

**FIGURE 1:**
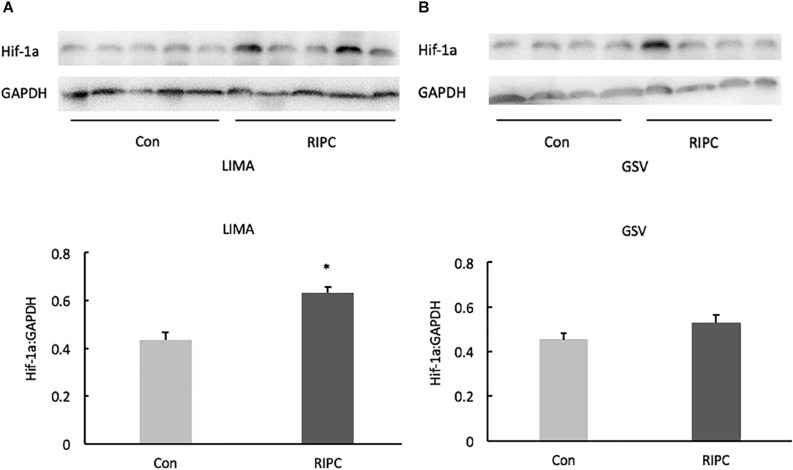
Hif-1α protein expression (Western blotting) in LIMA and GSV. **(A)** In LIMA samples, Hif-1 α protein expression in the RIPC group was higher than that in the control group. **(B)** In GSV samples, there was no significant difference of the Hif-1α levels in vein between RIPC and control group. GSV, great saphenous vein. LIMA, left internal mammary artery. *P < 0.05 vs. control group.

In the present study, RIPC group had a higher level of Hif-1α protein in LIMA samples when compared with control group (control vs. RIPC: 0.43 ± 0.04 vs. 0.63 ± 0.03, *p* < 0.05). However, the Hif-1α levels in vein were comparable in RIPC and control group (control vs. RIPC: 0.45 ± 0.03 vs. 0.53 ± 0.03, *p* > 0.05) (Figure 1).

## Discussion

In the present study treatment with remote ischemia preconditioning significantly shortens mechanical ventilation time and reduces myocardial damage by decreasing oxidative stress and reducing productions of inflammatory cytokines.

Compared with traditional coronary artery bypass grafting by pump, off-pump coronary artery bypass grafting has some advantageous effects, including shortening post-operation mechanical ventilation time, a decrease in cardiac complication, and improvement of patient’s recovery. However, myocardial injury is an unavoidable event of the surgery (Sellke et al., 2010). Regional ischemic “preconditioning” was first reported to reduce infarct size after transient occlusion of coronary artery (Ahmad et al., 2014). Later, remote preconditioning in limb is reported to attenuate myocardial injury in children undergoing congenital heart defect repair (Wu et al., 2018). However, the effectiveness of RIPC cardioprotection in adult patients undergoing cardiac surgery remains controversial. It is reported that RIPC reduces the release of myocardial enzymes after the cardiac surgery, but without clinical benefit (Ahmad et al., 2014; Benstoem et al., 2017; Xie et al., 2018). Thielmann et al. (2013) reported that RIPC provided perioperative myocardial protection and improved the prognosis of patients undergoing elective CABG surgery in a single-center randomized, double-blind, controlled trial. While another two large-scale, prospective, randomized, sham-controlled multi-center phase III trials (ERICCA and RIPHeart) show neutral results for both composite primary endpoints and troponin release by RIPC in patients underwent cardiac surgery under ischaemic cardiac arrest and cardiopulmonary bypass (Hausenloy et al., 2015; Meybohm et al., 2015). These inconsistent findings are probably related to differences in study protocols, confounding comorbidities, anesthetic regimens, and in surgical procedures, techniques, and protection regimens. The most plausible explanation for the lack of protection in ERICCA and RIPHeart is the use of propofol (more than 90% of patients in ERICCA and all patients in RIPHeart), a lipid-soluble anesthetic agent, eliminating reactive oxygen species (ROS) and interfering with the signal transduction pathway of RIPC somewhere upstream of STAT5 (Kottenberg et al., 2014), which is no more cardioprotcetive than volatile anesthesia such as isoflurane (Zaugg et al., 2014; Heusch and Gersh, 2016).

In the present study, patients in control group had elevated levels of cTnT, HFABP, and IMA, indicating that the surgery indeed induces myocardial damage. RIPC treatment reduced oxidative stress, decreased production of inflammatory cytokines, downregulated protein expression of myocardial injury makers, suggesting that RIPC protects surgery-induced damage in cardiac myocytes. Of note, RIPC also reduced the supportive ventilation time, confirming the beneficial effects of RIPC treatment in the process of the surgery (Azarfarin et al., 2014).

In the present study, RIPC treatment restored OPCABG-induced oxidative stress, suggesting that RIPC systemically reduces oxidative stress after the surgery. Enhanced local inflammation induces vasoconstriction and attracts lymphocytes (Kleinbongard et al., 2011). Kharbanda et al. (2001) has shown that RIPC confers cardioprotection by reducing neutrophil activation and endothelial dysfunction in patients with valve replacement surgery. In the present study, RIPC treatment reduced inflammatory cytokines such as IL-6, IL-8, and TNF-α levels, supporting the note that local ischemic conditioning associated with the inhibition of inflammatory responses confers cardiopretective effect (Kleinbongard et al., 2017). Anti-inflammatory cytokine IL-10 may be an important factor involved in the mechanistic pathway linking the remote organ to the heart. It is reported that RIPC by 3 cycles of 5 min ischemia and 5 min reperfusion on one hind limb induces late protection against myocardial IRI by increasing the expression of IL-10 (Cai et al., 2012). In the present study, RIPC treatment reduced production of pro-inflammatory cytokines and increased anti-inflammatory cytokine, which is consistent with an acute modification of inflammatory pathways in RIPC.

The transcription factor hypoxia-inducible factor (Hif)-1 is a central oxygen-sensitive player in the protective response to hypoxia (Ong and Hausenloy, 2012). Under normoxic condition, Hif-1β is constitutively expressed while Hif-1α is inactive. When responded to hypoxia, Hif-1α is up-regulated (Prabhakar and Overholt, 2000; Prabhakar and Semenza, 2012). It is reported that RIPC increased Hif-1α levels in cardiomyocytes of patients undergoing heart surgery (Albrecht et al., 2013). The mechanism of Hif-1α involved in cardioprotection remains unclear (Heusch, 2012). ROS plays an important role in cardioprotection under ischaemic preconditioning, and Hif-1α is a key factor in regulating cellular oxygen homeostasis (Lee et al., 2000; Semenza, 2012). During hypoxia, Hif-1α regulates the enzyme composition of the mitochondrial respiratory chain to alleviate ROS formation (Fukuda et al., 2007). But now Hif-1α is shown to be a pre-requisite for the mitochondrial ROS formation to initiate the protection by ischaemic preconditioning (Cai et al., 2008) In the present study, RIPC treatment upregulated Hif-1α protein expressions in the LIMA, but not GSV, suggesting that artery is more sensitive to hypoxia. Further study is required to investigate the role of Hif-1α for RIPC-induced cardioprotection in arteries.

### Study Limitations

Overall the present study was performed with a small amount of patients and in a single center. And patients recruited were relatively aged and often companied with other systemic diseases. These factors may affect the results and conclusion in the present study. The beneficial effects of preconditioning need to be further evaluated in long-term follow-up and in large-scale clinical trials. Some references show that the intravenous anesthetic propofol possesses antioxidant properties that could obscure the effects of RIPC. Thus, in our study, sevoflurane was used to maintain depth of anesthesia. As sevoflurane has been reported to have myocardial protection, whether it interferes with the protection effect of RIPC needs further study.

## Conclusion

Remote ischemic preconditioning protects off-pump coronary artery bypass grafting-induced cardiomyocytes damage. Better understanding of the underlying mechanism of the remote preconditioning is requested in further study.

## Ethics Statement

The present study was approved by Ethnics Committee, Zhongshan Hospital, Fudan University (https://ClinicalTrials.gov#NCT03340181).

## Author Contributions

HW, YH, YY, and KG designed the study. QL, LJ, and LX collected the clinical data and also managed the procedures in the Department of Anesthesiology. HW and YL wrote the manuscript, KG reviewed and revised the manuscript.

## Conflict of Interest Statement

The authors declare that the research was conducted in the absence of any commercial or financial relationships that could be construed as a potential conflict of interest.

## Footnotes

1 https://clinicaltrials.gov/#NCT03340181

## Abbreviations

cTnT: cardiac troponin T
GSV: great saphenous vein
HFABP: heart-type fatty acid binding protein
Hif: hypoxia-inducible factor
IMA: ischemia modified albumin
LIMA: left internal mammary artery
MDA: malondialdehyde
OPCABG: off-pump coronary artery bypass graft surgery
RIPC: remote ischemic preconditioning
ROS: reactive oxygen species.

## References

[B1] Ahmad A. M. Ali G. S. Tariq W. (2014). Remote ischemic preconditioning is a safe adjuvant technique to myocardial protection but adds no clinical benefit after on-pump coronary artery bypass grafting. *Heart Surg. Forum* 17 E220–E223. 10.1532/HSF98.2014391 25179977

[B2] Albrecht M. Zitta K. Bein B. Wennemuth G. Broch O. Renner J. (2013). Remote ischemic preconditioning regulates HIF-1alpha levels, apoptosis and inflammation in heart tissue of cardiosurgical patients: a pilot experimental study. *Basic Res. Cardiol.* 108:314. 10.1007/s00395-012-0314-0 23203207

[B3] Azarfarin R. Ashouri N. Totonchi Z. Bakhshandeh H. Yaghoubi A. (2014). Factors influencing prolonged ICU stay after open heart surgery. *Res. Cardiovasc. Med.* 3:e20159. 10.5812/cardiovascmed.20159 25785249 PMC4347792

[B4] Benstoem C. Stoppe C. Liakopoulos O. J. Ney J. Hasenclever D. Meybohm P. (2017). Remote ischaemic preconditioning for coronary artery bypass grafting (with or without valve surgery). *Cochrane Database Syst. Rev.* 5:CD011719 10.1002/14651858.CD011719.pub3 PMC6481544 28475274

[B5] Cai Z. Zhong H. Bosch-Marce M. Fox-Talbot K. Wang L. Wei C. (2008). Complete loss of ischaemic preconditioning-induced cardioprotection in mice with partial deficiency of HIF-1a. *Cardiovasc. Res.* 77 463–470. 10.1093/cvr/cvm035 18006459

[B6] Cai Z. P. Parajuli N. Zheng X. Becker L. (2012). Remote ischemic preconditioning confers late protection against myocardial ischemia-reperfusion injury in mice by upregulating interleukin-10. *Basic Res. Cardiol.* 107:277. 10.1007/s00395-012-0277-1 22752341 PMC3596418

[B7] Cheung M. M. Kharbanda R. K. Konstantinov I. E. Shimizu M. Frndova H. Li J. (2006). Randomized controlled trial of the effects of remote ischemic preconditioning on children undergoing cardiac surgery: first clinical application in humans. *J. Am. Coll. Cardiol.* 47 2277–2282. 10.1016/j.jacc.2006.01.066 16750696

[B8] Chowdhury U. K. Malik V. Yadav R. Seth S. Ramakrishnan L. Kalaivani M. (2008). Myocardial injury in coronary artery bypass grafting: on-pump versus off-pump comparison by measuring high-sensitivity C-reactive protein, cardiac troponin I, heart-type fatty acid-binding protein, creatine kinase-MB, and myoglobin release. *J. Thorac. Cardiovasc. Surg.* 135 1110.e1–1119.e10. 10.1016/j.jtcvs.2007.12.029 18455592

[B9] Fukuda R. Zhang H. Kim J. W. Shimoda L. Dang C. V. Semenza G. L. (2007). HIF-1 regulates cytochrome oxidase subunits to optimize efficiency of respiration in hypoxic cells. *Cell* 129 111–122. 10.1016/j.cell.2007.01.047 17418790

[B10] Hausenloy D. J. Candilio L. Evans R. Ariti C. Jenkins D. P. Kolvekar S. (2015). Remote ischemic preconditioning and outcomes of cardiac surgery. *N. Engl. J. Med.* 373 1408–1417. 10.1056/NEJMoa1413534 26436207

[B11] Hausenloy D. J. Mwamure P. K. Venugopal V. Harris J. Barnard M. Grundy E. (2007). Effect of remote ischaemic preconditioning on myocardial injury in patients undergoing coronary artery bypass graft surgery: a randomised controlled trial. *Lance.* 370 575–579. 17707752 10.1016/S0140-6736(07)61296-3

[B12] Heusch G. (2012). HIF-1a and paradoxical phenomena in cardioprotection. *Cardiovasc. Res.* 96 214–215. 10.1093/cvr/cvs145 22822099

[B13] Heusch G. (2018). 25 years of remote ischemic conditioning: from laboratory curiosity to clinical outcome. *Basic Res. Cardiol.* 113:15. 10.1007/s00395-018-0673-2 29516255

[B14] Heusch G. Botker H. E. Przyklenk K. Redington A. Yellon D. (2015). Remote ischemic conditioning. *J. Am. Coll. Cardiol.* 65 177–195. 10.1016/j.jacc.2014.10.031 25593060 PMC4297315

[B15] Heusch G. Gersh B. J. (2016). ERICCA and RIPHeart: two nails in the coffin for cardioprotection by remote ischemic conditioning? Probably not! *Eur. Heart J.* 37 200–202. 10.1093/eurheartj/ehv606 26508160

[B16] Kharbanda R. K. Peters M. Walton B. Kattenhorn M. Mullen M. Klein N. (2001). Ischemic preconditioning prevents endothelial injury and systemic neutrophil activation during ischemia-reperfusion in humans in vivo. *Circulation* 103 1624–1630. 10.1161/01.cir.103.12.1624 11273988

[B17] Kleinbongard P. Peters J. Jakob H. Heusch G. Thielmann M. (2018). Persistent survival benefit from remote ischemic preconditioning in patients undergoing coronary artery bypass surgery. *J. Am. Coll. Cardiol.* 71 251–262. 10.1016/j.jacc.2017.10.083 29325645

[B18] Kleinbongard P. Schulz R. Heusch G. (2011). TNF-alpha in myocardial ischemia/reperfusion, remodeling and heart failure. *Heart Fail. Rev.* 16 49–69. 10.1007/s10741-010-9180-8 20571888

[B19] Kleinbongard P. Skyschally A. Heusch G. (2017). Cardioprotection by remote ischemic conditioning and its signal transduction. *Pfluegers Arch.* 469 159–181. 10.1007/s00424-016-1922-6 27928644

[B20] Kottenberg E. Musiolik J. Thielmann M. Jakob H. Peters J. Heusch G. (2014). Interference of propofol with signal transducer and activator of transcription 5 activation and cardioprotection by remote ischemic preconditioning during coronary artery bypass grafting. *J. Thorac. Cardiovasc. Surg.* 147 376–382. 10.1016/j.jtcvs.2013.01.005 23465551

[B21] Lee S. H. Wolf P. L. Escudero R. Deutsch R. Jamieson S. W. Thistlethwaite P. A. (2000). Early expression of angiogenesis factors in acute myocardial ischemia and infarction. *N. Engl. J. Med.* 342 626–633. 10.1056/NEJM200003023420904 10699162

[B22] Mahesh B. Choong C. K. Goldsmith K. Gerrard C. Nashef S. A. Vuylsteke A. (2012). Prolonged stay in intensive care unit is a powerful predictor of adverse outcomes after cardiac operations. *Ann. Thorac. Surg.* 94 109–116. 10.1016/j.athoracsur.2012.02.010 22579949

[B23] Meybohm P. Bein B. Brosteanu O. Cremer J. Gruenewald M. Stoppe C. (2015). A multicenter trial of remote ischemic preconditioning for heart surgery. *N. Engl. J. Med.* 373 1397–1407. 10.1056/NEJMoa1413579 26436208

[B24] Ong S. G. Hausenloy D. J. (2012). Hypoxia-inducible factor as a therapeutic target for cardioprotection. *Pharmacol. Ther.* 136 69–81. 10.1016/j.pharmthera.2012.07.005 22800800

[B25] Prabhakar N. R. Overholt J. L. (2000). Cellular mechanisms of oxygen sensing at the carotid body: heme proteins and ion channels. *Respir. Physiol.* 122 209–221. 10.1016/S0034-5687(00)00160-2 10967345

[B26] Prabhakar N. R. Semenza G. L. (2012). Adaptive and maladaptive cardiorespiratory responses to continuous and intermittent hypoxia mediated by hypoxia-inducible factors 1 and 2. *Physiol. Rev.* 92 967–1003. 10.1152/physrev.00030.2011 10.1152/physrev.00030.2011 22811423 PMC3893888

[B27] Przyklenk K. Bauer B. Ovize M. Kloner R. A. Whittaker P. (1993). Regional ischemic “preconditioning” protects remote virgin myocardium from subsequent sustained coronary occlusion. *Circulation* 87 893–899. 10.1159/000108686 7680290

[B28] Sakpal T. V. (2010). Sample size estimation in clinical trial. *Perspect. Clin. Res.* 1 67–69. 21829786 PMC3148614

[B29] Sellke F. W. Chu L. M. Cohn W. E. (2010). Current state of surgical myocardial revascularization. *Circ. J.* 74 1031–1037. 10.1253/circj.cj-10-0321 20467145 PMC4761444

[B30] Semenza G. L. (2012). Hypoxia-inducible factors in physiology and medicine. *Cell* 148 399–408. 10.1016/j.cell.2012.01.021 22304911 PMC3437543

[B31] Thielmann M. Kottenberg E. Kleinbongard P. Wendt D. Gedik N. Pasa S. (2013). Cardioprotective and prognostic effects of remote ischaemic preconditioning in patients undergoing coronary artery bypass surgery: a single-centre randomised, double-blind, controlled trial. *Lancet* 382 597–604. 10.1016/S0140-6736(13)61450-6 23953384

[B32] Wu Q. Wang T. Chen S. Zhou Q. Li H. Hu N. (2018). Cardiac protective effects of remote ischaemic preconditioning in children undergoing tetralogy of fallot repair surgery: a randomized controlled trial. *Eur. Heart J.* 39 1028–1037. 10.1093/eurheartj/ehx030 28329231 PMC6018784

[B33] Xie J. Zhang X. Xu J. Zhang Z. Klingensmith N. J. Liu S. (2018). Effect of remote ischemic preconditioning on outcomes in adult cardiac surgery: a systematic review and meta-analysis of randomized controlled studies. *Anesth. Analg.* 127 30–38. 10.1213/ANE.0000000000002674 29210794

[B34] Xue M. Xu H. (2017). Summary of the forum on integration of traditional Chinese and Western medicine. *Chin. J. Integr. Tradit. West. Med.* 10:1270.

[B35] Zaugg M. Lucchinetti E. Behmanesh S. Clanachan A. S. (2014). Anesthetic cardioprotection in clinical practice from proof-of-concept to clinical applications. *Curr. Pharm. Des.* 20 5706–5726. 10.2174/1381612820666140204120829 24502570

